# Wound-Induced Endogenous Jasmonates Stunt Plant Growth by Inhibiting Mitosis

**DOI:** 10.1371/journal.pone.0003699

**Published:** 2008-11-11

**Authors:** Yi Zhang, John G. Turner

**Affiliations:** School of Biological Sciences, University of East Anglia, Norwich, United Kingdom; Max Planck Institute for Developmental Biology, Germany

## Abstract

When plants are repeatedly injured their growth is stunted and the size of organs such as leaves is greatly reduced. The basis of this effect is not well-understood however, even though it reduces yield of crops injured by herbivory, and produces dramatic effects exemplified in ornamental bonsai plants. We have investigated the genetic and physiological basis of this “bonsai effect” by repeatedly wounding leaves of the model plant Arabidopsis. This treatment stunted growth by 50% and increased the endogenous content of jasmonate (JA), a growth inhibitor, by seven-fold. Significantly, repeated wounding did not stunt the growth of the leaves of mutants unable to synthesise JA, or unable to respond to JA including *coi1*, *jai3*, *myc2*, but not *jar1*. The stunted growth did not result from reduced cell size, but resulted instead from reduced cell number, and was associated with reduced expression of *CycB1;2*. Wounding caused systemic disappearance of constitutively expressed JAZ1::GUS. Wounding also activates plant immunity. We show that a gene, *12-oxo-phytodienoate reductase*, which catalyses a step in JA biosynthesis, and which we confirm is not required for defence, is however required for wound-induced stunting. Our data suggest that intermediates in the JA biosynthetic pathway activate defence, but a primary function of wound-induced JA is to stunt growth through the suppression of mitosis.

## Introduction

Plants are sessile and many are adapted to survive unfavourable growth conditions, including cold, salinity, drought and a variety of biotic stresses including herbivory. Environmental stresses such as these are estimated to reduce plant growth and crop yield by approximately 22% worldwide [1]. Herbivory by insects reduces plant growth [2], and herbivory by mammals can produce extremely stunted “bonsai” plants [3]. Growth-inhibition is also caused by other abiotic stresses besides wounding, including salinity, cold, and drought. Salinity-induced growth inhibition of Arabidopsis appears to result from the absence of gibberellin (GA)-mediated growth promotion, involving the stabilization of DELLA proteins that inhibit growth [4]. The growth regulators, ethylene and abscisic acid also inhibit growth apparently by stabilizing DELLA proteins [4], [5]. Interestingly, the inhibition of growth by ethylene is via the stimulation of growth-inhibitory concentrations of auxin in the root elongation zone [6], [7]. Therefore, although low concentration of auxin promotes root growth by destabilizing DELLA proteins [8], high concentrations of auxin induced by ethylene evidently suppress root growth by stabilizing the DELLA proteins.

The jasmonates (JAs) are also produced in plants exposed to biotic or abiotic stresses, including wounding. These are a group of oxylipin plant signaling molecules that are synthesised from chloroplast linolenic acid [9], [10]. Topical application of JAs to plants has profound effects on both growth and physiology: the treated plants become stunted and the leaves and roots in particular are reduced in size [11]–[13]. In addition, plants treated with JAs show increased synthesis of secondary products [14] and have enhanced resistance to attack by pests and pathogens [10]. These physiological effects are associated with transcriptional reprogramming of 10% or more of the genome, involving both the enhancement and the inhibition of transcription of different genes [15], [16]. There was considerable overlap between the genes activated by wounding and by JA, supporting the key role for JA in the plant wound response. JA responses may also be regulated independently of wounding. For example, JA formation in stamens is independent of wounding [17]. In addition, the microRNA, miR319 controls the level of TCP transcription factors which, in turn, activate lipoxygenase 2 , required for JA synthesis [18].

**Figure 1:**
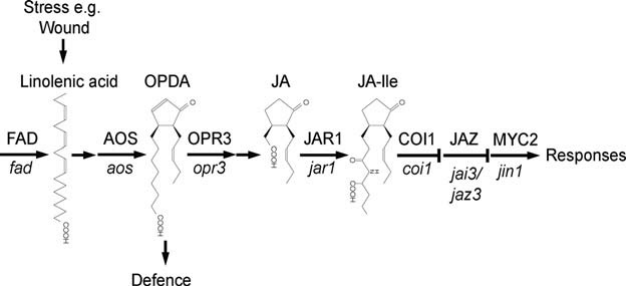
JA and the wound signal pathway. Linolenic acid synthesised by fatty acid desaturases (FAD) is released from chloroplast lipid in response to a stress such as wounding or attack by pests and pathogens, and it serves as substrate for jasmonate (JA) biosynthesis. 12-oxophytodienoic acid (OPDA) is sufficient for defence against pests and pathogens. Key enzymes in JA synthesis are allene oxide synthase (AOS) and 12-oxophytodienoate reductase 3 (OPR3). Lower case gene symbols indicate mutants used in this study. JAR1 couples JA to an amino acid which activates a signal pathway, involving COI1, which also contributes to defence. Specific wound responses are activated by a COI1-dependent signal that removes JAZ proteins that suppress the transcription factor MYC2/JIN1, leading to its activation.

Many of the functions of JAs in plants have been revealed by the study of mutants defective in their biosynthesis and signalling. Some of the key genes in JA biosynthesis and JA signaling for which mutants are available are shown in Figure 1. In Arabidopsis, plants carrying mutations in each of the three omega-3 fatty acid desaturases *fad3-2fad7-2fad8*, fail to synthesise JA, are male sterile and have dramatically reduced resistance to pests and pathogens [17], [19]. Significantly topical application of JA to the *fad3-2fad7-2fad8* mutant restores defence and fertility. Similarly, the *aos* mutant fails to synthesise JA, is male sterile, and its fertility is restored by JA [20]. Although the *opr3* mutant is also male sterile and its fertility is restored by application of JA, it is resistant to attack by fungal pathogens and insect pests [21], [22]. This observation suggested that the substrate for the enzyme 12-oxo-phytodienoate reductase 3 (OPR3), OPDA, is a signalling molecule able to induce plant defences though not able to promote pollen development [21], [22].

Progress towards an understanding of how the perception of JA leads eventually to the reprogramming of the plant genome has come through the isolation of genes defined by mutants insensitive to JA-induced growth inhibition, including *JAR1*, *COI1*, *JAZ1*, *JAI3*(*JAZ3*), and *JIN1*(*MYC2*) [23]–[27]. As a consequence of stress-induced JA biosynthesis, the JAR1 protein couples JA to one of a number of amino acids including isoleucine. The JA-amino acid conjugate activates binding of members of a new family of proteins, the JAZ proteins, to an SCF complex containing the F box protein COI1, resulting in their proteasome-mediated destruction. The JAZ proteins bind and repress at least one transcription factor, MYC2, that activates a subset of JA responsive genes when the JAZ proteins are degraded [25], [26].

Although one of the most dramatic effects of exogenously applied JA on plants is to stunt growth, the role of endogenous JAs as natural regulators of plant growth has not been widely studied, nor do we understand the mechanism by which JA reduces growth. Previous work has shown that the *aos* mutant, defective in JA biosynthesis, shows less wound-induced growth inhibition than wild type plants, providing evidence that endogenous JAs stunt growth of wounded plants [28], [29]. Here we present evidence that wounding leads to the production of JA that suppresses growth by inhibiting mitosis in young leaves and meristems through a mechanism that involves COI1, JAZ and MYC2, but apparently not JAR1.

## Results

### Wounding activates JA synthesis, JAZ1 destruction, and JA responses

**Figure 2:**
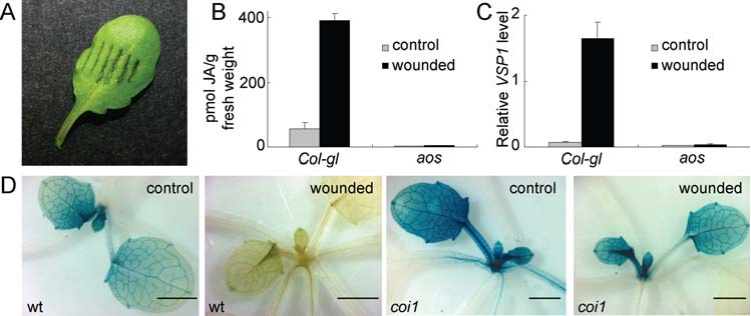
Effect of wounding on Arabidopsis plants. (A) Leaves were wounded by applying pressure with forceps having serrated teeth, bruising the tissue. For JA determination (Figure 2B) and the wound-induced growth inhibition experiments (Figure and Figure 4), the largest leaf was wounded on each occasion. No leaf was wounded more than once. At the end of the experiment, plant usually had 14–16 leaves, of which 10 were wounded (the size of the wounded leaves varied). (B) JA content in control and wounded *Col-gl* and *aos* plants. (C) Quantitative RT-PCR was used to measure expression of *VSP1* in untreated controls and wounded *Col-gl* and *aos* plants. The *VSP1* level was relative to *ACTIN2*. (D) Eighteen-day-old wild type *Col-gl* and *coi1-1* seedlings containing the *35S*::*JAZ1*::*GUS* transgene were untreated as control or wounded on a single leaf. GUS activity was detected histochemically one hour afterwards. The wounded leaf is not shown in the images.

We have used a simple and reproducible wounding treatment to study the effect of wound-induced JA on growth. Leaves were wounded with tweezers, the serrated teeth of which produced 4–6 bruises across the width of the leaves (Figure 2A). Twenty-one-day old *Col-gl* and *aos* plants were wounded as in Figure 2A. For each plant, a total of ten leaves were wounded, one leaf per day, over a period of ten days. To confirm that this treatment activates JA synthesis, one and a half hours after the last wound, the plants were harvested and the content of JA was measured. JA increased more than seven-fold to 391 pmol/g in wounded wild type leaves, but was present at less than 5 pmol/g in untreated controls and the wounded *aos* mutant (Figure 2B). We examined whether this wounding treatment activated JA responses. Two and a half hours after the wound, expression of the JA-responsive and COI1-dependent gene *VSP*1 [30] was found to be increased in two week old *Col-gl* plants, but not in the *aos* plants (Figure 2C). We also tested whether other components of the JA signal pathway were involved in this response. Plants containing a *JAZ1*::*GUS* transgene under the control of the constitutively expressed 35S promoter in a wild type and *coi1-1* background were wounded on a single leaf. GUS activity declined within 60 minutes of wounding wild type plants, but was not degraded to the same extent in *coi1* plants. The wounded leaf is not within the image in Figure 2D, which therefore indicates that the wound signal is systemic, rapid, and reaches the shoot meristem. Control plants containing the 35S::*JAZ1::GUS* transgene in the *coi1*-1 mutant background had higher activity than those containing the transgene in the *Col-0* background, suggesting that endogenous JA suppresses the recombinant protein.

### Wounding reduces growth of wild type plants but not JA mutants

**Figure 3:**
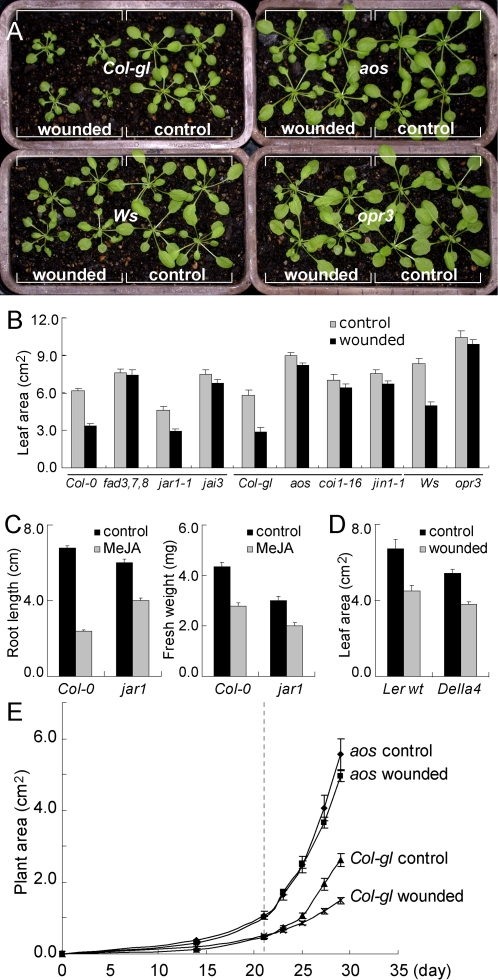
Effect of wounding on growth of wild type plants and JA mutants. (A) Thirty-one-day old wild type, *aos* (in *Col-gl* background), and *opr3* (in *Ws* background) plants after wounding. Controls are unwounded. (B) Leaf area of unwounded and wounded 31-day-old wild type plants and JA mutants (n≥10). Lines underneath the histogram indicate the same genetic background, *Col-0*, *Col-gl*, or *Ws*. (C) Effect of MeJA on root length and fresh weight of wild type *Col-0* and *jar1* plants (n≥25). (D) Leaf area of unwounded and wounded 31-day-old wild type *Ler* and quadruple-DELLA mutant plants (n≥10). (E) Growth rates of control and wounded *Col-gl* and *aos* plants. Wounding of plants started on day 21, indicated by the dotted line (n = 12).

Wounding increases the OPDA [31] and JA content of plant tissue, and application of JA suppresses growth. To test whether wound-induced JA affects growth, we examined mutants deficient in JA synthesis and JA response. For this, ten leaves of each plant were wounded, one leaf per day over a period of ten days. At the end of this treatment, leaf area and the fresh weight of the above ground part were measured. Wounding significantly reduced the size and fresh weight of *Col-gl* plants (P<0.001) but not of the *aos* mutant in the *Col-gl* background (Figure 3A, Figure 3B and Figure S1) as reported previously [28]. During the preparation of this manuscript it was also reported by Yan et al 2007 that repeated wounding did not stunt growth of the *aos* mutant as much as in wild type plants [29].Wounding also significantly reduced the growth of the wild type (*Ws*) plants (P<0.001), but not the *opr3* mutant in the *Ws* background. Similarly, leaf area and fresh weight of the *fad3-2fad7-2fad8* triple mutant, defective in JA biosynthesis, was not significantly reduced by wounding compared to its parent. Mutants in the JA signal pathway including *coi1-16* [32], *jai3/jaz3* and *jin1-1/myc2* were also not significantly reduced in size by wounding compared to their parents. However growth of the *jar1-1* [33] mutant was inhibited by wounding (P<0.001), and its final size was not significantly different from its parent wild type plant, *Col-0* (Figure 3B). *jar1-1* was originally identified because its root growth was not inhibited as much as the wild type in the presence of 10 µM MeJA [33]. We confirmed that on media containing 10 µM MeJA root growth of *jar1-1* was inhibited 33% (P<0.001) whereas that of the parent, *Col-0*, was inhibited 65% (P<0.001) (Figure 3C). However, reduction in leaf growth, as determined by reduction in fresh weight of *jar1-1* and *Col-0* growing on 10 µM MeJA was 33% and 37% respectively (Figure 3C). This indicates that MeJA was more inhibitory to *jar1* leaf growth than root growth. This is consistent with reports that *jar1* has wild type wound response in leaf tissue [34], [35]. The control (unwounded) mutants in JA biosynthesis (*fad3-2fad7-2fad8*, *aos*, and *opr3*) and JA signaling (*jai3*, *coi1*-16 and *jin1*) were larger (Figure 3B) and had greater fresh weight (Figure S1) than their corresponding control parental plants.

Together, these results indicate that endogenous JA significantly suppresses growth in untreated wild type plants, and that wound-induced JA significantly suppresses growth further. Plants combining mutations in four genes for DELLA proteins (RGL1, RGL2, GAI, RGA) were insensitive to growth inhibition by salt [4]. However, growth of these plants was inhibited by wounding to the same extent as wild type plants (Figure 3D). We tested mutants in other signal pathways for salicylic acid (*npr1*), abscisic acid (*aba1*), auxin (*aux1*, *axr1*), and ethylene (*ein2*, *etr1*). All showed significant wound-induced growth inhibition, compared to unwounded controls, in the range of 22%–49% (P<0.05, Figure S2). This indicates that the signal pathways defined by these mutants are not required for wound-induced growth inhibition. Tobacco seedlings showed wound-induced growth inhibition of 47% (P<0.001), indicating that this other plant species is also stunted by wounding (Figure S3).

### Wounding inhibits growth rate but not cell size

Responses to JA are typically rapid and we therefore examined the time course of growth inhibition by wounding. Twenty-one-day old *Col-gl* and *aos* plants were wounded on nine successive days, each day on a different leaf, and the whole plant area was measured from digital images of growing plants taken every other day (Figure 3E). Plant area of untreated *Col-gl* was significantly less than that of untreated *aos* (P<0.001) from day 14 to day 29. Plant area of wounded *Col-gl* plants was significantly less than that of the controls at 4, 6 and 8 days after the first wound (P<0.05, P<0.001, P≪0.001, respectively), but the total leaf number was not significantly different from that of unwounded plants. On the wounded *Col-gl* plant, growth of both wounded and unwounded leaves was reduced. By contrast, there was no significant difference between the growth rate of control and wounded plants of the *aos* mutant. Wounding therefore inhibited growth rate of wild type plants within 4 days, but had no significant effect on growth rate of the *aos* mutant.

Because wound-induced JA reduced the rate of leaf expansion we tested whether JA also affected the rate of leaf emergence. Twenty-one day old plants were sprayed with MeJA (100 µM) at 2-day intervals for 10 days, and the appearance of new leaves over that period was recorded. These treatments with MeJA did not significantly reduce the rate of leaf emergence (Table 1).

**Table 1:** Effect of methyl jasmonate (MeJA) on the rate of leaf emergence.

Leaf emergence/day	control	MeJA	p-value (two-tail, t-test)
*Col-gl*	0.873±0.072	0.768±0.060	0.380
*aos*	0.965±0.112	0.861±0.064	0.439
*jar1*	0.714±0.075	0.533±0.085	0.150
*coi1*	0.906±0.074	0.807±0.026	0.207
*jin1*	0.840±0.126	0.793±0.061	0.746

To investigate whether wound-induced suppression of growth arises from a reduction in cell size, cell number, or both, twenty-one-day old wild type and *aos* plants were wounded over a ten-day period as before, and cleared in chloral hydrate. The area of leaf 8 from each plant was measured, and the cross-sectional area of palisade mesoplyll cells in leaf 8 was measured with the aid of a microscope. Although the size of *Col-gl* leaves was reduced more than two-fold by the wounding, the cross-sectional area of the palisade mesophyll cells was not significantly different from the unwounded controls (Table 2). Leaves of the *aos* mutant were larger than leaves of *Col-gl* plants, and were not reduced in size by wounding. Interestingly, palisade mesophyll cells of the *aos* mutant had a smaller cross sectional area than of the *Col-gl* plants (Table 2). Evidently, the smaller size of wounded *Col-gl* leaves was due to a smaller number of cells and not to a reduction in the size of the cells. By contrast, the *aos* mutant leaves not only were larger but also contained more and smaller cells than those of the *Col-gl* parent.

**Table 2:** Effect of wounding on the size of leaf 8 and cell size.

	*Col-gl*		*aos*	
	unwounded	wounded	unwounded	wounded
Leaf 8 area cm^2^	0.58±0.01^a^	0.26±0.02^b^	0.68±0.06^c^	0.68±0.02^c^
Cell size µm^2^	1770±103^a^	1890±210^a^	1540±216^b^	1260±127^c^
cell number/leaf(×10^−4^)	3.28±0.24	1.37±0.23	4.41±0.78	5.38±0.42

Each value in the table is the mean±SE of the measurements. For each row, means with different superscript letters are significantly different, p<0.05.

### Wounding and MeJA reduces cell division

**Figure 4:**
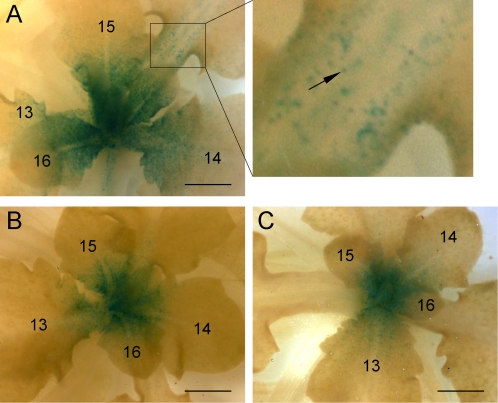
Histochemical detection of GUS in 31-day-old wild type *Col-0* plants containing the *CycB1;2*::*GUS* reporter. (A) Untreated plants as control. Inset is an enlarged part of the petiole to indicate the individually stained cells (arrow). (B) Plants wounded on ten successive days. (C) Plants sprayed three times over 10 days with MeJA (100 µM) Numbers indicate leaf number. Scale bar, 1 mm.

**Figure 5:**
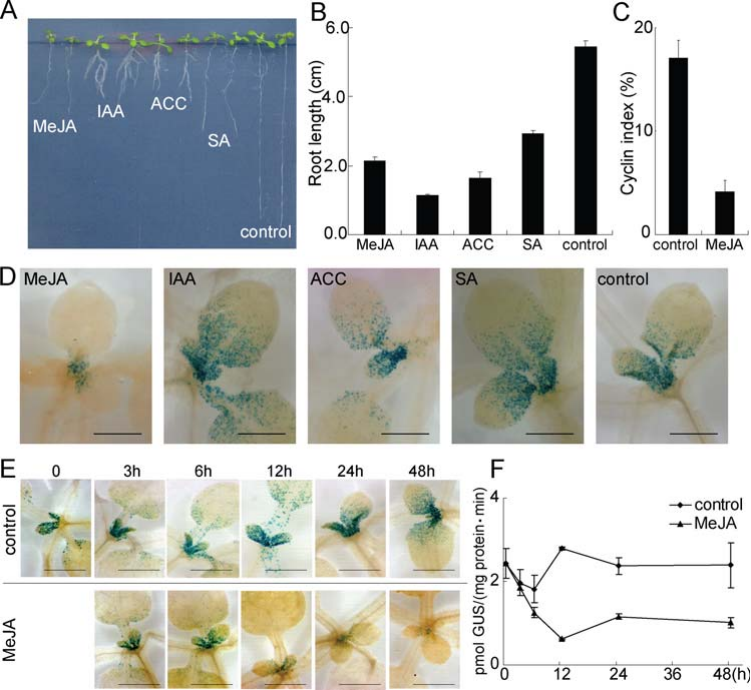
Effect of MeJA, IAA, ACC, and SA on growth and GUS expression in wild type *Col-0* plants containing *CycB1;2*::*GUS* transgene. (A) Four-day-old seedlings were transferred to MS as control or to MS containing MeJA (50 µM), IAA (2 µM), ACC (10 µM) or SA (50 µM) for 10 days. (B) Root length of 14 days old seedlings growing on media containing MeJA, IAA, ACC, and SA. (C) Cyclin index of control and MeJA treated plants from (A). (D) Histochemical detection of GUS in seedlings from (A). Scale bar, 0.5 mm. (E) Twelve-day-old seedlings grown on MS media were transferred to MS as control or to MS containing 50 µM MeJA. GUS was detected histochemically at the indicated intervals after transfer. (F) Twelve-day-old seedlings grown on soil were sprayed with water as control or 500 µM MeJA, and again at the intervals at which samples were taken. GUS activity in the samples was measured by the MUG assay. Data points are means of three samples of 60 seedlings each.

Results in Table 2 suggested that wounding may reduce cell division in leaves. To test this, we used an Arabidopsis line containing the *pCycB1;2* gene fused to GUS as a reporter for cell division [36]. Twenty-one-day old plants containing the *CycB1;*2::*GUS* transgene were wounded on ten successive days, each day on a different leaf, or sprayed with MeJA (100 µM) on three successive occasions at three day intervals. On day 31, plants were harvested and GUS activity was detected by histochemical staining. GUS activity was associated with single cells and strongly restricted to the basal half of the developing leaf (Figure 4A), as previously described [36]. Significantly, GUS activity was reduced in plants that had been wounded (Figure 4B) or treated with MeJA (Figure 4C). This is consistent with the results presented in Table 2, indicating that wounding and MeJA reduce leaf growth by reducing cell division. Other compounds that inhibited growth, including ACC, SA, and IAA (Figure 5A and Figure 5B) were examined for an effect on mitosis. Whereas JA reduced the cyclin index by more than 75% (Figure 5C and Figure 5D), IAA, ACC and SA caused no detectable reduction in the cyclin index (Figure 5D).

It takes approximately 12 hours for an Arabidopsis cell to complete mitosis. We therefore treated seedlings with MeJA and determined the time-course of reduction in activity of the CyclinB::GUS reporter. Twelve-day-old seedlings containing the *CyclinB1;2::GUS* transgene were transferred to media containing 50 µM MeJA, and seedlings were removed at intervals and GUS was detected histochemically (Figure 5E). GUS activity had virtually disappeared from the shoot meristem and young leaves by 12 hours. To quantify the effect of MeJA on the GUS activity, 12-day old soil-grown seedlings containing the *CyclinB1;2::GUS* transgene were sprayed at intervals with MeJA (500 µM), seedlings were harvested at intervals, and GUS was measured spectrophotometrically using the MUG assay (Figure 5F). GUS activity was detectably reduced within 6 hours, and maximally reduced by 12 hours.

### OPDA does not suppress growth

**Figure 6:**
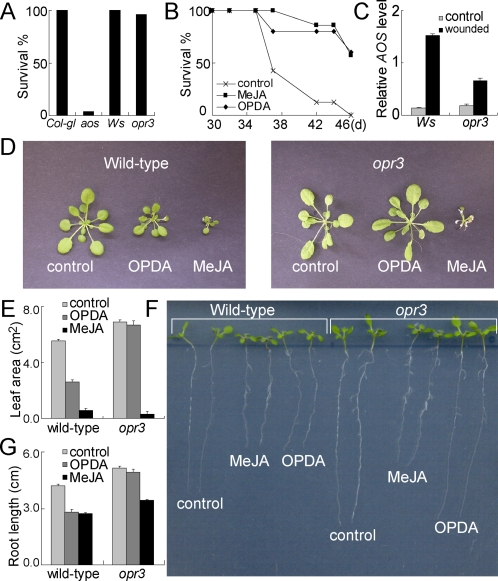
JA and OPDA in defence against *Bradysia impatiens*, and growth inhibition. (A) Survival of *Col-gl*, *aos*, *Ws* and *opr3* plants grown in soil infested with larvae of *Bradysia impatiens*. Adult flies were introduced at day 14 and survival was counted on day 46. (B) Survival of *aos* plants grown in soil infested with larvae of *Bradysia impatiens* as (A) and sprayed with MeJA (100 µM), or OPDA (100 µM), or water as a control at day 30, 32 and 35. (C) Quantitative RT-PCR was used to measure the expression of *AOS* in *Ws* and *opr3* plants untreated as control or wounded twice on two leaves. The *AOS* level was relative to *ACTIN2*. (D) OPDA (100 µM) or MeJA (100 µM) was applied to leaves of wild type and *opr3* plants at days 21, 23 and 25. Images are of plants at 31 days. (E) Leaf area of plants from (D). (F) Five-day-old wild type *Ws* and *opr3* plants were transferred to MS as control and MS containing 10 µM MeJA or 10 µM OPDA. Images were taken 7 days after transfer. (G) Length of roots of seedlings from (F) (n≥21).

Both JA and OPDA have been reported to activate Arabidopsis defences [19], [22]. We confirmed that the *opr3* mutant survived an attack by *Bradysia* which, we show here, reduced the population of the *aos* mutant to 4%, (Figure 6A). Application of JA or OPDA to the *aos* mutant enhanced its survival of attack by *Bradysia* (Figure 6B). *AOS*, a gene required for OPDA and JA synthesis (Figure 1), is transcriptionally activated by JA and by wounding [15]. We demonstrate that wounding activated the transcription of *AOS* in both wild type (*Ws*) and the *opr3* mutant (Figure 6C). Because JA inhibits plant growth [11]–[13], we tested whether OPDA also inhibited growth. Leaves of twenty-one-day old wild type and *opr3* plants were wetted with 100 µM MeJA, 100 µM OPDA, or water as control, on three successive occasions at two day intervals, and leaf area was measured 4 days after the final treatment. Both OPDA and MeJA reduced leaf area of wild type plants, and OPDA reduced the leaf area of wild type plants but not of the *opr3* plants(Figure 6D and Figure 6E). Similarly, MeJA inhibited root growth of wild type plants and the *opr3* plants, and OPDA inhibited root growth of wild type plants but not of the *opr3* plants (Figure 6F and Figure 6G). Evidently, OPDA is not as growth-inhibitory as MeJA, and the reduced leaf area and root length of wild type plants treated with OPDA is likely due to its conversion to JA or one of its metabolites [37].

## Discussion

When plants are repeatedly wounded their growth is reduced, and in extreme examples their leaves are markedly smaller, as revealed in bonsai trees. We show here that repeated wounding of Arabidopsis reduced growth and that this was mediated through wound-induced production of the growth inhibitor, JA. The principal evidence for this was that Arabidopsis mutants unable to synthesise JA (*fad3-2fad7-2fad8*, *aos* and *opr3*), or insensitive to JA-induced growth inhibition (*coi1*, *jai3*, *jin1*), had significantly less wound-induced growth inhibition than their wild type parents. These results indicate that the “bonsai effect” induced by repeated wounding is not a direct consequence of, for example, the physiological disorder that would undoubtedly occur in the injured tissues, but is a consequence of the plant sensing injury and activating the synthesis of JA which, in turn, inhibits growth. The wounded JA synthesis mutants and signaling mutants were consistently- though not significantly-smaller than the unwounded mutants. This may be due to release of other wound-induced inhibitors, including the phytoprostanes [38]. We report here that the quadruple-DELLA mutant showed wild-type growth inhibition by wounding (Figure 3E). Evidently therefore, the JA and the DELLA stress-response signal pathways regulate different types of stress-induced growth inhibition.

The time course of the wound- and JA-responses reported here are consistent with our understanding of the sequence of events in the pathway. Following a wound JAZ1::GUS disappeared in a *COI1*-dependent manner in 1 h; the JA responsive *VSP1* increased within 2.5 hours; and leaf growth was inhibited within 4 days. Direct application of JA also caused disappearance of JAZ1::GUS within 1 h.

The reduced size of wounded leaves reported here was not accompanied by detectable reduction in size of the cells. Likewise bonsai trees have leaves that are drastically reduced in size but the leaf cells are the same size as, or larger than, cells of normal-sized tree leaves [39]. The reduced size of wounded or MeJA-treated leaves was associated with a reduction in mitotic index, as revealed by the *CycB1;2* reporter. Direct application of JA reduced the mitotic index within 6 h. We observed that expression of the *CycB1;2* reporter was confined to cells in the shoot apical meristem, leaf primordia, and to the basal half of young leaves approximately 0.5 mm in breadth as previously reported [36]. The wound treatment we used to suppress the cyclin index was applied to mature leaves in which there was little or no evidence of mitosis. We conclude that a systemic signal must move from the wounded tissue and travel to the shoot apex where mitosis is reduced. We observed also that a single wound to a mature leaf eliminated the JAZ1::GUS protein from the shoot apex and surrounding tissues of plants containing the *35S*::*JAZ1*::*GUS* transgene. Together, these results provide compelling evidence that wound-induced JA activates the destruction of JAZ protein and the suppression of mitosis.

The reduced size of cytokinin deficient plants containing the *35S*:*CKX* transgene was associated with a smaller apical meristem, and was attributed to either reduced division or earlier differentiation of cells in the apical meristem [40]. We observed that the rate of leaf initiation in plants treated with MeJA was not significantly reduced compared to untreated plants. This suggested therefore that MeJA did not alter the rate of leaf determination. It is possible that JA reduces the size of the founding population of cells prior to leaf determination, as well as reducing cell division during leaf development as we show here, or both. This is consistent with Swiatek et al [41], [42], who found that JA arrested cell division at the G2 phase in tobacco BY2 cell cultures, and this was associated with reduction in B type cyclin dependent kinases, and a reduction in expression of *CycB1;1*. We also observed that mutants deficient in JA synthesis (*fad3-2fad7-2fad8*, *aos* and *opr3*) were larger than their corresponding parents. The *aos* mutant palisade mesoplyll cells were smaller and more numerous than of the parent. Together, these observations suggest that in unwounded plants endogenous JA reduces cell division and leaf size, and that the smaller size of repeatedly-wounded plants is due to enhanced production of JA and further reduction in cell division. JAs also reduce mitosis of animal cells, and have anticancer activity, although the mechanism remains uncertain [43].

**Figure 7:**
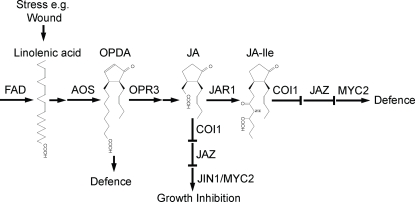
JA and the wound-induced growth inhibition pathway. COI1, JAZ proteins and MYC2 -but not JAR1- are required for wound-induced growth inhibition. This model branches the signal pathway at JA.

The *jar1* mutant was different from the other JA signal mutants *coi1*, *jai3* and *myc2* in that it did display wound-induced stunting. Consistent with this, *jar1* was the only one of the JA mutants examined in this study that was smaller than its parental wild type. Although *jar1* was isolated for its JA-insensitive root growth inhibition [33], its shoot fresh weight was significantly inhibited by JA. Apparently therefore, JAR1 is required for some JA responses including root growth inhibition [33] and defence against pathogens [44]–[48], but not for the wound response [35] and, we show here, the wound-induced leaf growth inhibition. Our model for JA signalling therefore recognizes the apparent difference between JA-dependent defence and JA-dependent wound response (Figure 7). A point of uncertainty arises however because it has been shown recently that wounded *jar1* produced approximately 10% of the wound-induced JA-isoleucine conjugate compared to wild-type plants [35], possibly synthesized by an enzyme other than JAR1. This may be sufficient to produce the wild-type wound-response in *jar1*. Although we can conclude that JAR1 is not required for the wound-induced stunting we report here, we therefore cannot conclude whether or not JA-ILE is involved in the wound-induced inhibition of leaf growth.

It has been suggested that OPDA, an intermediate in JA synthesis, is in fact the active signalling molecule in plant response to stress [49]. Certainly, topical application of OPDA induces expression of both JA regulated genes as well as genes that are not regulated by JA [50]. Genes for JA and OPDA biosynthesis are transcriptionally activated by JA and by wounding [15]. We report that *AOS*, one of the genes involved in OPDA synthesis, was also activated by wounding the *opr3* mutant, indicating the possibility that OPDA production can occur in the absence of JA. We confirm a previous report that OPDA is apparently sufficient for defence against the fungus gnat larvae, *Bradysia impatiens* [22]. However, we also show that wound-induced growth inhibition is not mediated by OPDA, but requires JA or a compound after the step that is catalysed by OPR3. Therefore we propose that OPDA can activate defence but does not significantly suppress growth, whereas JA can suppress growth and may also activate defence.

In conclusion, we show here that endogenous and wound-induced jasmonate suppresses cell division and, thereby, growth of leaves. Presumably there is a selective advantage to wound-induced stunting: possibly this response allows the plant to complete its life cycle with fewer cells requiring fewer resources.

## Materials and Methods

### Chemicals, Plants Materials and Growth Condition

Chemicals were from Sigma-Aldrich, with the following exceptions: 12-oxo-phytodienoic acid (OPDA) (Cayman Chemical, USA), methyl JA (MeJA) (Bedoukian Research, USA), and Silwet (LEHLE SEEDS, USA). Mutants and wild-type lines were from the cited authors. Arabidopsis wild type and mutants plants were grown at 22°C, with 8 hours of white fluorescent light at 100 µmolm^−2^ s^−1^ on soil or on 0.8% agar media containing half-strength Murashige and Skoog (MS) salts and 0.5% sucrose. Leaf area was measured with Windias equipment and software (DeltaT, UK). To measure growth rate of plant size, images of growing plants were taken on day 14, day 21 and every second day thereafter until day 31, and plant area was measured by image analysis software (http://www.comp.leeds.ac.uk/yanong/alm). To investigate the effect of MeJA on leaf emergence rate, wild-type or mutant lines (n≥14) were grown on soil. Treatment with 100 µM MeJA started from day 21, then every second day until day 31, and the total number of leaves of each seedling was recorded. The daily leaf emergence rate was estimated from the number of new leaves detected every second day between day 21 and day 31. To determine the effect of MeJA and OPDA on root growth, wild-type plants and *opr3* mutants were grown on media for 5 days. Seedlings with equal root length (n = 21) were selected and transferred to media containing 10 µM MeJA, 10 µM OPDA or MS only as control (each contained 0.3% ethanol). Seven days later, root length was measured.

### JA Measurement

*aos* and wild-type *Col-gl* plants weighing approximately 1 g were harvested in triplicate, ground in liquid nitrogen, and the powder was resuspended in 5 ml methanol and incubated at 4°C overnight. The suspensions were clarified at 1.36×10^3^ g for 5 min and the supernatants were dried with nitrogen gas. The residues were washed twice in 90 µl methanol and 600 µl diethyl ether, and the washes combined and the solutions were clarified at 2.15×10^3^ g for 5 min. NH_2_ columns (Phenomenex, USA) were washed with 2 ml diethyl ether, and the samples were applied. The column was then washed twice with 0.8 ml (chloroform: isopropanol, 2∶1.7) and samples were eluted with diethyl ether containing 2% acetic acid and dried with nitrogen gas. JA content was determined by LCMS.

### RNA extraction and Real-Time PCR

Triplicate samples (each containing 8 seedlings) were collected 90 min after wounding. RNA was extracted from the whole plant using the RNeasy Plant Mini kit with the optional DNase digestion step following the manufacture's protocol (Qiagen, UK). RNA was quantified (Nanodrop ND1000 spectrophotometer, NanoDrop Technologies, USA) and cDNA was synthesised from 1 µg of the extracted RNA with SUPERSCRIPTII RNase Reverse Transcriptase (Invitrogen, UK). Quantitative PCR was performed in 96-well optical plates in an ABI 7700 Sequence Detection System (TaqMan; PerkinElmer). Each well contained 5 ng of the synthesised cDNA, 15 µl 2×TaqMan universal PCR mastermix (PE Applied Biosystems), 100 nM probe, 200 nM of each primer, and water to a final volume of 25 µl. Thermocycler conditions comprised an initial holding at 50°C for 120 sec then 95°C for 10 min. This was followed by a two-step TaqMan PCR program consisting of 95°C for 15 sec and 60°C for 60 sec, for 40 cycles. The Arabidopsis *ACTIN2* gene (AT3G18780) was chosen as a normalisation standard for TaqMan analysis of *VSP1* gene (AT5G24780) and *AOS* gene (AT5G42650). The sequences of the primers and the cDNA specific probe for *ACTIN2* were those described previously [51]. Specific primers and fluorogenic probes for *VSP1* and *AOS* were designed using Primer Express 3.0 software (PE Applied Biosystems), and were synthesized by Sigma-Aldrich, the sequences of which were:

*VSP*1-F, 5′-TCTCAAGGCTGTTGGTGTAACAA-3′;
*VSP1*-R, 5′-TTGTACACCACTTGCGTCAACTT-3′;
*VSP1*-probe, 5′-ATGGAAGCATCTCATACTCAAGCCAAACGG-3′;
*AOS*-F, 5′-CGAATCATATCGCCGGAAA-3′;
*AOS*-R, 5′-ATGCGCCGGTAAGGATTTT -3′;
*AOS*-probe, 5′-TCTCAATCACAAACAACCTCGCCACCA-3′.

### Insect Infestation

Arabidopsis *opr3* (in the *Ws* background), *aos* (in *Col-gl* background) mutants and their parental wild types (n = 48, each) were grown on soil under 10 hours of light at 22°C, 120 µmolm^−2^ s^−1^. Fourteen-day-old plants were transferred to ventilated chambers, and seven batches of 30 to 50 adult *Bradysia impatiens* flies were introduced every other day into each chamber. Surviving plants were recorded from day 30 to day 46.

### Cell Measurement

After the final wounding on day 31, leaf 8 was collected from ten unwounded and ten wounded plants of *Col-gl* and *aos*. Leaf tissues were fixed in Carnoy's fixative (ethanol: glacial acetic acid, 3∶1) overnight at 4°C, transferred to 100% ethanol to remove chlorophyll, rehydrated, and transferred to chloral hydrate: water: glycerol solution, 8∶2∶1. Images of the leaf palisade mesophyll cell layer were taken, 25% and 50% above the leaf base and from the leaf tip, and the cross-section area of palisade cells (n>150) were measured by Zeiss Axiocam 4.5 software (Carl Zeiss, Germany).

### Detection of GUS Activity

Plants containing the *CycB1;2*::GUS transgene were wounded or treated with MeJA, and GUS was detected histochemically [36]. To quantify cyclin index, wild type seedlings containing the *CyclinB1;2*::GUS reporter were grown on media for 4 days and seedlings of similar size (n = 40) were transferred to media containing 50 µM MeJA, 2 µM IAA, 10 µM ACC, 50 µM SA or MS only, as control, for another 10 days. The cyclin index was determined by the ratio between the numbers of GUS stained cells and the total number of cells in an area of approximately 0.02 mm^2^ 25% above the leaf base of leaf 4. To further investigate how rapidly JA may have an effect on mitosis, seedlings containing the *CycB1;2*::*GUS* reporter were grown on media for 12 days before they were transferred to media containing 50 µM MeJA or MS only as control, and samples were collected at 0 h, 3 h, 6 h, 12 h, 24 h and 48 h after transfer, and GUS was detected histochemically. To quantify cyclin activity, 12-day-old seedlings containing the *CycB1;2*::*GUS* reporter grown on soil were sprayed with 500 µM MeJA, or water as control, at 0, 3 h, 6 h, 12 h, 24 h and 48 h, and triplicate samples (each containing 60 seedlings) were collected immediately before each of these treatments. GUS activity was determined with 4-methylumbelliferyl-D-glucuronide (MUG) according to [52] and was expressed as pmol MU per mg protein per min. Protein concentrations were measured by Bradford assay [53].

## Supporting Information

Figure S1 Effect of wounding on fresh weight of Arabidopsis plants. Plants were wounded as described in Fig. 2A, 31-day-old wild type plants and JA mutants (n≥10) were harvested, and their fresh weights were determined. Bars indicate SE.
(0.36 MB TIF)

Figure S2 Effect of wounding on leaf area of *aba1*, *npr1*, *etr1*, *ein2*, *aux1* and *axr1* plants. Plants were wounded as described in Fig. 2A. Leaf area reduction of the 31-day-old plants was measured (n≥10).
(0.08 MB TIF)

Figure S3 Effect of wounding on growth of *Nicotiana benthamiana* plants. (A) Twenty-day-old tobacco plants were wounded once by bruising with forceps as described in Fig 2A, and this was repeated on each of seven successive days. Twenty-nine-day-old control unwounded and wounded plants are shown. (B) Leaf area of unwounded and wounded 29-day-old tobacco plants.
(7.43 MB TIF)
