# Supplementary figures and images for: Wound-Induced Endogenous Jasmonates Stunt Plant Growth by Inhibiting Mitosis
Source: PLoS One. 2008 Nov 11;3(11):e3699. doi: 10.1371/journal.pone.0003699 (PMC2577035; doi:10.1371/journal.pone.0003699)

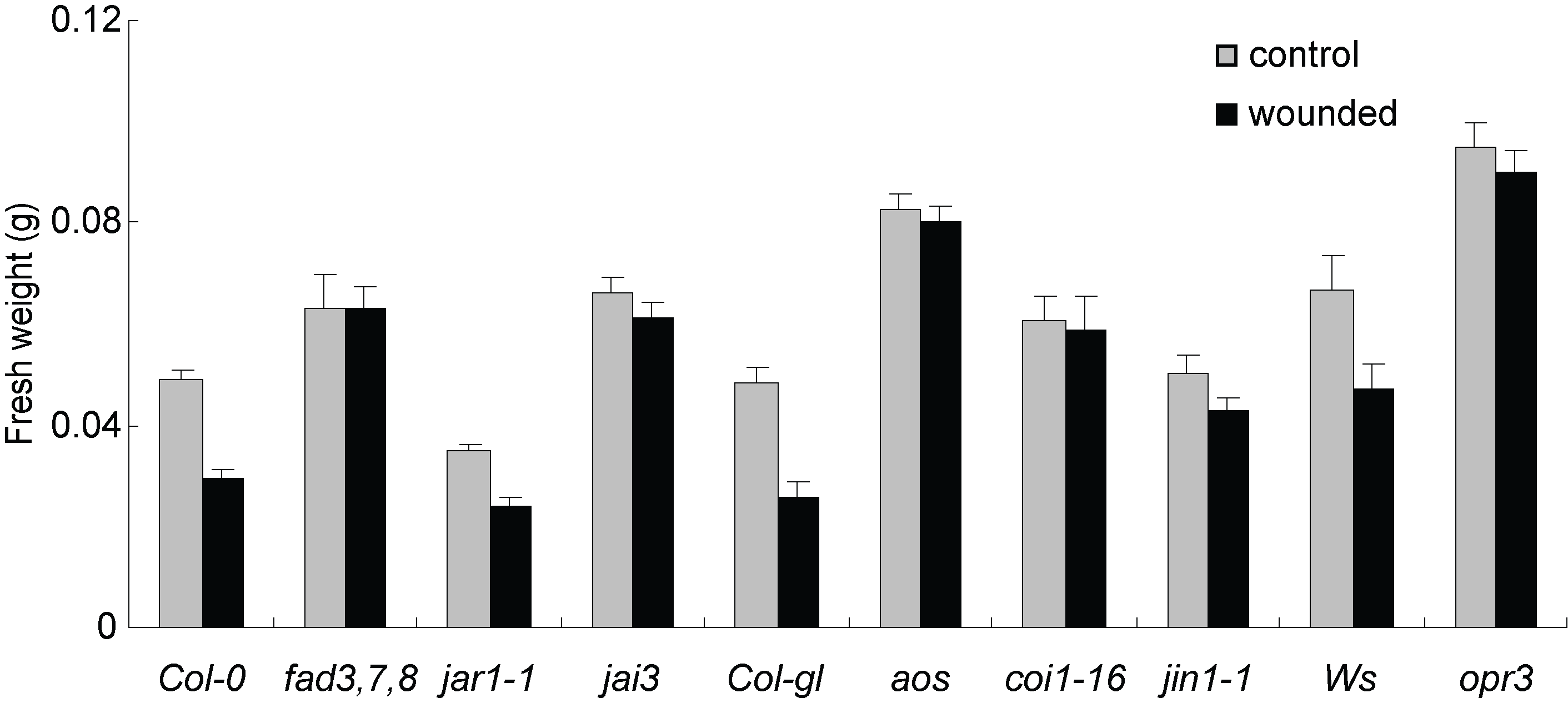

Supplement: Figure S1 — Effect of wounding on fresh weight of Arabidopsis plants. Plants were wounded as described in Fig. 2A, 31-day-old wild type plants and JA mutants (n≥10) were harvested, and their fresh weights were determined. Bars indicate SE. (0.36 MB TIF) [file pone.0003699.s001.tif]

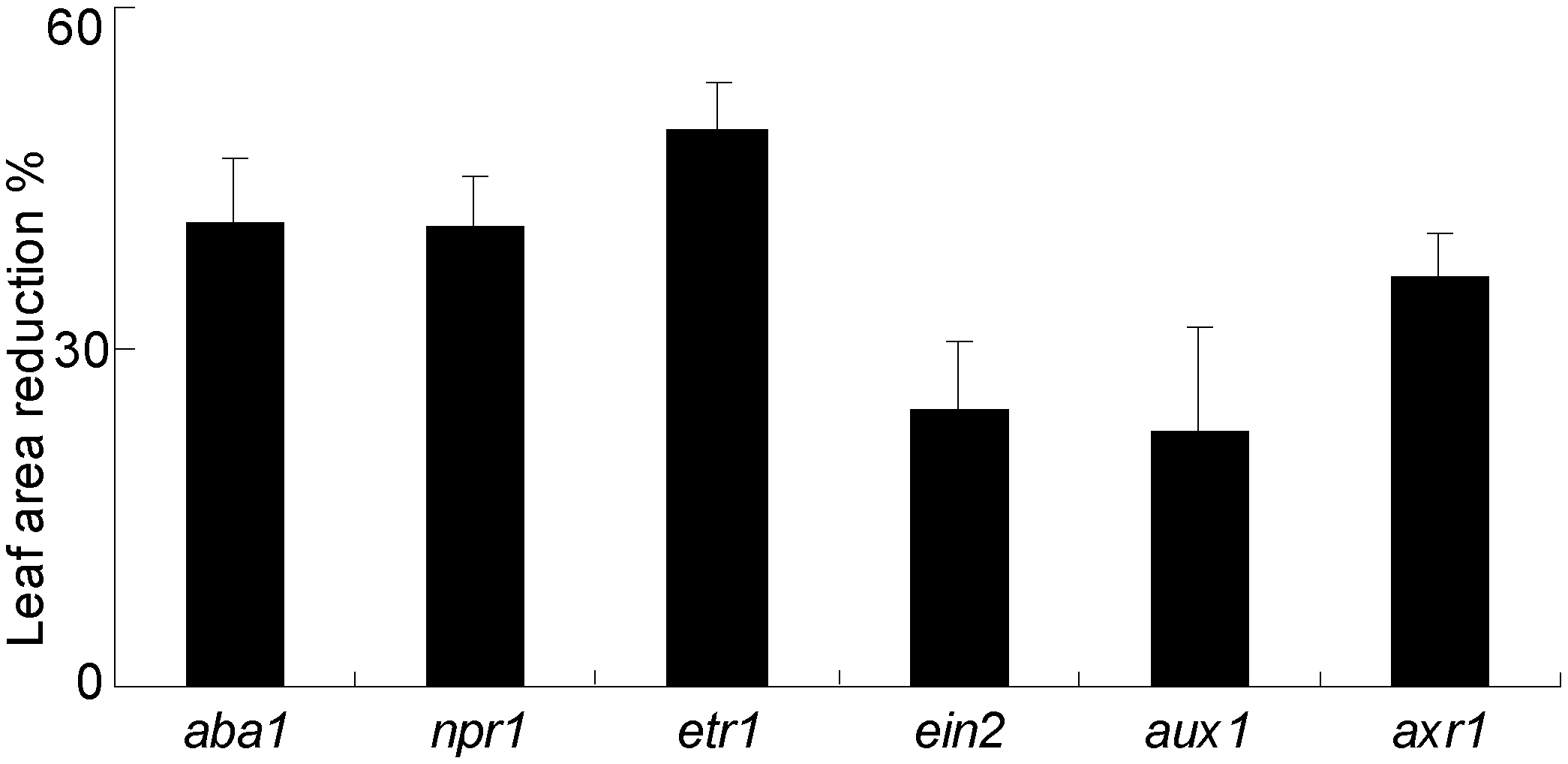

Supplement: Figure S2 — Effect of wounding on leaf area of aba1, npr1, etr1, ein2, aux1 and axr1 plants. Plants were wounded as described in Fig. 2A. Leaf area reduction of the 31-day-old plants was measured (n≥10). (0.08 MB TIF) [file pone.0003699.s002.tif]

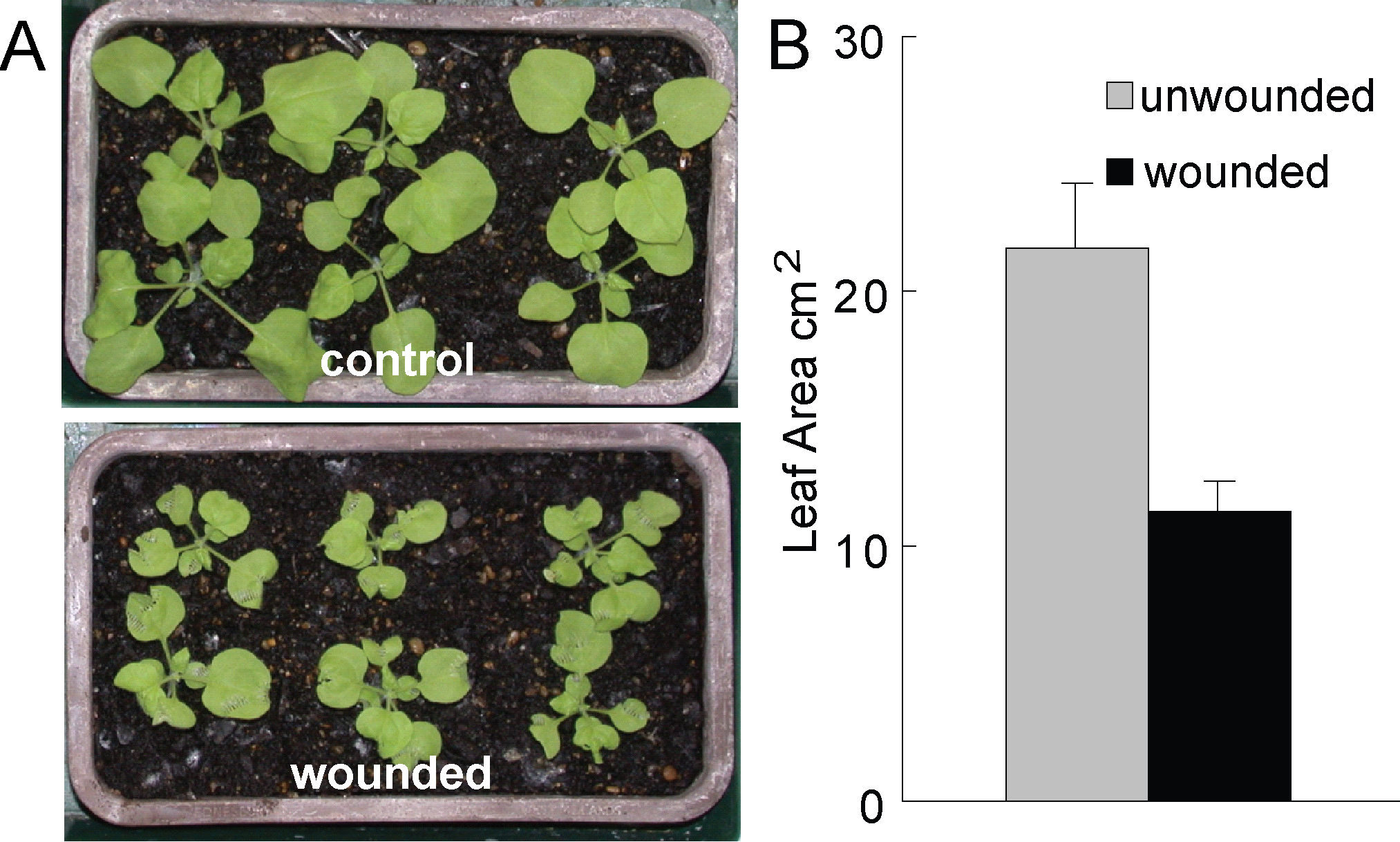

Supplement: Figure S3 — Effect of wounding on growth of Nicotiana benthamiana plants. (A) Twenty-day-old tobacco plants were wounded once by bruising with forceps as described in Fig 2A, and this was repeated on each of seven successive days. Twenty-nine-day-old control unwounded and wounded plants are shown. (B) Leaf area of unwounded and wounded 29-day-old tobacco plants. (7.43 MB TIF) [file pone.0003699.s003.tif]
